# Antibacterial, anti-inflammatory and peroxidase-mediated cyclooxygenase-1 inhibitory properties of *Fusarium solani* extract

**DOI:** 10.1080/13880209.2019.1606260

**Published:** 2019-06-04

**Authors:** Kenneth Ngwoke, Nwalusiuka Tochukwu, Chinechem Ekwealor, Valerie Nwankwo, Uju Obi-Okafor, Chisom Izundu, Festus B. C. Okoye, Charles Esimone, Peter Proksch, Chen Situ

**Affiliations:** a Faculty of Pharmaceutical Sciences, Nnamdi Azikiwe University, Awka, Nigeria;; b Institut für Pharmazeutische Biologie und Biotechnologie, Heinrich Heine Universitat, Dusseldorf, Germany;; c Institute for Global Food Security, School of Biological Sciences, Queen's University Belfast, Belfast, UK

**Keywords:** COX-1, MIC, inflammation, soil fungi, pathogens, cancer

## Abstract

**Context:** Nigerian soil fungi population is unexplored. It is hypothesized that they harbour new bioactive chemicals. This hypothesis is based on the large percentage of currently approved medicines that originated from soil-inhabiting micro-organisms

**Objectives:** To investigate the antimicrobial and anti-inflammatory properties of *Fusarium solani* ethyl acetate (EtOAc) extract selected based on its broad spectrum of antimicrobial potential in an overlay experiment with seven other soil fungi strains.

**Materials and methods:** Fungus number 6 (F6), identified by molecular characterization as *Fusarium solani* (Mart.) Sacc (Nectriaceae) was selected for studies from eight purified soil fungi due to its superior broad-spectrum antibiotics producing potential following agar overlay experiment. F6 was fermented for 21 d and the minimum inhibitory concentration (MIC) of its EtOAc fermentation extract (dose range: 12.5–100 µg/mL) was determined using agar dilution method for *Staphylococcus aureus*, *Bacillus subtilis*, *Pseudomonas aeruginosa*, *Escherichia coli*, *Salmonella typhi* and anti-inflammatory properties determined using rat-paw (250–500 mg/kg) and xylene induced oedema (250–500 µg/kg) (in Swiss albino rats and mice) models, respectively. The ability of the extract to inhibit cyclooxygenase (COX) enzyme was also determined *in vitro* using Cayman test kit-760111.

**Result:** The MIC of the EtOAc extract was <12.5 µg/mL for *S. aureus*, *P. aeruginosa* and *Escherichia coli*. It inhibited xylene induced oedema by 65% compared with 61% observed for diclofenac and was significantly (*p* < 0.05) better than diclofenac in rat-paw-oedema model within the first phase of inflammation. The extract inhibited COX-1 peroxidase-mediated activities with an IC_50_ below 5 µg/mL.

**Conclusions:** The extract exhibited strong antibacterial and anti-inflammatory properties, warranting further investigations into therapeutic potential of this fungus. This study design can be adapted in soil fungi metabolomic investigations. We report for the first time the potent anti-inflammatory property of the ethyl acetate extract of soil strain of *F. solani* with a possible mechanism of action that involves the inhibition of COX enzyme.

## Introduction

The serendipitous discovery of penicillin from *Penicillium notatum* Westling (Trichocomaceae) heralded an era of anti-infective discovery from microbial sources. With the continuous development and emergence of resistant strains of pathogenic organisms, especially in the Gram-negative organisms that have innate resistant capabilities (Tillotson 2016), the continuous effort in new antimicrobial discovery will remain relevant until a lasting solution is found.

Other pharmacologically important compounds, such as antilipidemic agents were subsequently discovered from microorganisms. Lovastatin, an antilipidemic compound, was isolated from *Aspergillus terreus* Thom (Trichocomaceae) (Endo 2010) and its derivatives have been isolated from *A. sclerotiorum* Huber Sp080903f04 (Phainuphong et al. 2016). Actinomycin D, doxorubicin and bleomycin are some other examples of therapeutic drugs derived from microorganisms currently used in the treatment of cancers (Orlikova et al. 2014).

The effective management of all types of cancer has been an unmet health need to date. Various approaches to cancer management have also been adopted by different healthcare systems worldwide. Recently, a link between inflammation and cancer progression has been established (Roxburgh and McMillan 2014) so that compounds with anti-inflammatory properties are proposed to have potential use in cancer treatment and prevention. It is foreseen that the use of anti-inflammatory agents as adjunct therapy will be a common approach in cancer management in clinical practice in the near future (Chan and Detering 2013; Schafer and Kaschula 2014).

*Fusarium solani* (Mart.) Sacc (Nectriaceae) is a soil fungus popularly known for its mycotoxigenic properties (Shi et al. 2017) and as a causative pathogen of skin and nail infections (Kuruvilla and Dias 2012). Many bioactive compounds have been reported from the extracts of various *Fusarium* species including some potent antibacterial compounds isolated from the endophytic strain of *F. solani* (Kyekyeku et al. 2017*)*. Also, potent cytotoxic properties against various cancer cell lines and significant inhibitory activity against *Leishmania braziliensis* have been reported for the endophytic strain of *F. oxyporum* W.C. Snyder & H.N. Hans (Hyphomycetes) (Nascimento et al. 2012). In addition, an unrelated investigation of *F. oxyporum* for bioactive metabolites resulted in the discovery of antinematodal constituents within this species (Bogner et al. 2017). Furthermore, two other bioactive compounds, fusapyrone and deoxyfusapyrone isolated from the rice culture of *Fusarium semitectum* Berkeley & Ravenel (Nectriacae) have been found to display various levels of antimicrobial activities against plant and human pathogens (Altomare et al. 2000).

In this work, the antimicrobial and anti-inflammatory properties of *Fusarium solani* ethyl acetate (EtOAc) extract were investigated. We report for the first time the potent anti-inflammatory property of the ethyl acetate extract of soil strain of *F. solani* with a possible mechanism of action that involves the inhibition of cyclooxygenase (COX) enzyme.

## Materials and methods

### Sample collection

Approximately 100 g of soil was collected aseptically from a spot around the electric power generating plant in the Faculty of Pharmaceutical Sciences, Nnamdi Azikiwe University, Awka-Nigeria in March 2017. A sterile spatula was used to collect the soil samples from a layer 10 cm below the surface after scraping the surface. The sample was placed in a sterile sample container and was immediately taken to the Pharmaceutical Microbiology & Biotechnology Laboratory where it was analysed.

### Isolation and purification of soil fungi

The soil fungi were isolated by the soil dilution technique described by Waksman (1922). Briefly, the soil sample (1 g) was suspended in 10 mL of double distilled water to make a microbial suspension. Serial dilutions of this suspension (10^−1^–10^−5^) were then prepared. Dilutions 10^−2^ and 10^−4^ were introduced into different labelled sterile Petri-dishes (triplicate of each dilution). Molten Malt Extract Agar (MEA) (Oxoid, Basingstoke, UK), previously sterilized at 121 °C, 15 psi for 15 min and cooled to 45 °C was added and swirled evenly to ensure the homogeneity of the mixture and also to make the colonies discrete. Chloramphenicol (50 mg/L) was added to MEA medium to prevent bacterial growth. The Petri dishes were left to set for 15 min and then incubated at room temperature for 7 d in the dark.

### Determination of antibiotic production by the fungal isolates

A method reported by Chen et al. (2012) was used to determine the antibiotic production potential of the fungal isolates against *E. coli*, *Pseudomonas aeruginosa*, *Bacillus subtilis*, *Salmonella typhi*, *Staphylococcus aureus*, *A. niger*, and *C. albicans*. The agar culture of each pure fungal isolate was aseptically cut into a rectangular shape and placed on Mueller-Hinton agar previously inoculated with the different test organisms. This was then incubated at 37 °C for 24 h, after which the zone of inhibition of each test organism from the overlayed fungal agar was measured. The experiment was carried out in triplicate. (NB: To determine which fungal culture to study, the antimicrobial sensitivity determination was carried out using the CLSI prescribed method.)

### Identification of fungal cultures

The selected fungus (F6) was identified according to a molecular biology protocol which includes DNA amplification and sequencing of the ITS region, described previously by Ebrahim et al. (2016). The identification was carried out by Dr. Peter Eze, a pharmaceutical microbiologist of the Institute of Pharmaceutical Biology, Heinrich Heine University, Dusseldorf, Germany. The sequence has been deposited in the GenBank with accession number MG772815.

### Fermentation of pure fungal isolate

The solid fermentation medium was prepared by autoclaving 100 g of local rice with 200 mL of distilled water in 1 L conical flask at 121 °C for 15 min and this was allowed to cool to room temperature. Thereafter, segments were aseptically cut from the actively growing pure isolates on MEA and inoculated onto the fermentation medium contained in 1 L Erlenmeyer flask. This was properly sealed, and the fermentation process was allowed for 21 d at 30 °C under static conditions.

### Extraction of secondary metabolites

The fermentation was stopped with the addition of 500 mL of EtOAc. The solid medium was cut into small sections using a sterile spatula and the mixture was agitated intermittently for 2 d and then filtered using Whatman No. 1 filter paper. The filtrate was concentrated at 40 °C under reduced pressure using a rotary evaporator. The corresponding extract was weighed after evaporation, and the dried fungal extract was stored at 4 °C until needed.

### High-performance liquid chromatography (HPLC) analysis

The extract was subjected to HPLC analysis for preliminary dereplicative identification of its components. Exactly 1 mg/mL of the extract was prepared in HPLC grade methanol (Sigma Aldrich, Hamburg, Germany), sonicated for 10 min and centrifuged at 3000 rpm for 5 min. A 1:5 serial dilution was carried out to obtain a final concentration of 0.2 mg/mL solution and 20 µL of which was analysed in a Dionex HPLC system equipped with photodiode array detector (UVD340s, Dionex Softron GmbH, Germering, Germany) using 125 mm Eurosphere-10 C18 prefilled column (Knauer, Berlin, Germany) with 4 mm internal diameter and 5 µm particle size. The mobile phase comprised nano-pure water adjusted to pH 2 with formic acid and methanol. Separation was monitored at 254 nm and peaks were identified by dereplication.

### Determination of minimum inhibitory concentrations (MIC) (agar dilution)

The MIC of the extracts was determined for each of the test organisms using agar dilution method recommended by Clinical Laboratory Science Institute (CLSI 2017). Stock solutions of 2 mg/mL (2,000 µg/mL) of extract were prepared. Then, 2-fold serial-dilution was carried out to get a range of concentrations (1000, 500, 250 and 125 µg/mL). Thereafter, 10-fold dilution was obtained for each of the concentrations. Exactly 1 mL from each of these concentrations was transferred into sterile Petri dishes and 9 mL of molten agar cooled to 45 °C was added resulting to final concentrations of 100, 50, 25 and 12.5 µg/mL, respectively. The mixture was rocked clockwise and anticlockwise to ensure proper mixing. Following that, a loopful each of the test organisms previously standardized to McFarland turbidity was streaked on the solidified agar. A Petri dish containing only 1 mL of DMSO (Sigma Aldrich, Hamburg, Germany) mixed with the sterile molten agar and the organisms were prepared as above to serve as the negative control. The culture plates were then incubated at 37 °C for 24 h. After incubation, the plates were examined for visible microbial growth. The plates were observed for another 7 d.

### Experimental animals

The animals (Swiss albino rat and mice) were housed in standard laboratory conditions of 12 h light, room temperature, and 40–60% relative humidity. They were allowed access to food (Guinea feeds Nigeria Ltd, Awka, Nigeria) and water. All animal experiments were conducted in compliance with NIH guide for care and use of laboratory animals (National Institute of Health [NIH] (2011) pub No: 85–23), following the protocol approved by the Ethical Committee on the Use of Animal in Research of Nnamdi Azikiwe University.

### Determination of anti-inflammatory activity using rat paw oedema model

Anti-inflammatory activity of the ethyl acetate extract was carried out according to the method of Okokon et al. (2012) and Kim et al. (2017) with slight modification in doses and time. A total of 168-week old Swiss albino rat models (equal number of males and females) were grouped into 4 groups of 4 rats per group. Group 1 received 10 mg/kg of distilled water, group 2 received 1.66 mg/kg of diclofenac [dose determined using km factor calculation (Shin et al. 2010)], group 3 received 250 mg/kg of extract, group 4 received 500 mg/kg of extract. One hour after the administration, inflammation was induced by single sub-plantar injection of 0.1 mL raw egg albumin, then inflammation was checked at 30 min, 1, 2, 3 and 4 h post administration for the paw size. The % inhibition was calculated using the following formula:

$$\text{\% Inhibition = }\frac{A-B\;}{A}\;\times \;100,$$

where *A* = Difference in control (Sterile water group), *B* = Difference in treatment group.

### Anti-inflammatory test using xylene-induced oedema model

A total of 166-week old Swiss albino mice models (equal number of males and females) were grouped into 4 groups of four animals each. Group 1 was given equivalent volume of the vehicle (5% Tween 80) on the posterior part of the left ear. Group II received 500 µg/ear of diclofenac gel. Group III received 250 µg/ear of the extract while group IV received 500 µg/ear of the extract. About 10 min after treatment, inflammation was induced by topical administration of 10 µL of xylene at the anterior part of the treated ear. Two hours after the induction, the animals were sacrificed by cervical dislocation. A Cork borer of 6 mm diameter was used to punch out a section of both the treated and the untreated ears. The weight of each ear was determined using an analytical weighing balance. Anti-inflammatory activity was calculated as percentage inhibition of oedema using the formula:

$$\%\;\mathrm{Inhibition}=\frac{\left\{\begin{array}{l} \text{Difference in ear weight }\left(\mathrm{control}\right)\;\\ -\text{ difference in ear weight }(\text{test substance}) \end{array}\right\}}{\text{Difference in ear weight }(\mathrm{control})}\times \;100$$

### Acetic acid-induced writhing experiment

Analgesic activity (stomach writhing) was studied using the method employed by Ezeja et al. (2011). A total of 16 mice were used and they were grouped into 4 groups of 4 mice each. The animals were starved for 12 h prior to the experiment, having access to drinking water alone. The animals were given the treatment regimen as follows: Group 1 received 10 mL/kg of 5% Tween 80 orally (negative control) whereas group 2 received 15 mg/kg diclofenac [dose determined using km factor calculation (Shin et al. 2010)]. Group 3 was given orally 250 mg/kg extract and group 4 received 500 mg/kg extract. After 30 min of administration of the drugs, pain was induced by a single intraperitoneal injection of 0.1 mL/10 g 1% (v/v) acetic acid. The number of constrictions (writhes) for each mouse was counted for 30 min commencing from 5 min after the intraperitoneal injection of acetic acid. The percentage writhing inhibition was calculated using the formula: %Inhibition *=*$\frac{C-T}{T}$*×* 100, where c = number of writhes in negative control group and T = number of writhes in treatment group.

### Cyclooxygenase (COX-1 and COX-2) inhibition assay

A stock solution of *F. solani* fermentation extract was prepared in methanol as 1 mg/mL concentration. Test concentrations of 25, 50 and 100 µg/mL were prepared by serial dilution. The extract was tested in triplicates using a commercial COX (Ovine) colourimetric inhibitory screening assay kit following the instructions recommended by the manufacturer (Cayman test kit-760111, Cayman Chemical Company, Ann Arbor, MI) (George et al. 2014). The COX (ovine) inhibitor screening assay measures the peroxidase activity of COX which is assayed colourimetrically by monitoring the appearance of *N*,*N*,*N*′,*N*′-tetramethyl-*p*-phenylenediamine. Diclofenac (MW = 296.14) was used as the positive control for inhibition of COX-1 and COX-2. A mixture of 10 μL of test extract in 0.1 M Tris–HCl pH 8.0 (assay buffer), 150 µL assay buffer, 10 µL Heme and 10 µL of enzyme (COX-1or COX-2) in the inhibitor wells were pre-incubated with the enzyme at 25 °C for 10 min prior to the addition of arachidonic acid (AA). The reaction was initiated by addition of 20 μL of 10 mM AA after the addition of 20 µL of colourimetric substrate and the plate was incubated at 25 °C for another 2 min. Plate was read at 590 nm in ELISA plate reader. The reading was repeated at intervals of 2–5 min for over 30 min to determine the reaction rate and the apparent IC_50_ value.

## Statistical analysis

The results were analysed using Microsoft Excel version 2016 and presented as mean ± standard error of mean (SEM). Significance difference between negative control and extract treated groups were determined using one-way analysis of variance (ANOVA) at *p* < 0.05 while *post-hoc* Turkey test was used to determine differences between treatments. Two-way ANOVA was applied on the COX assay result.

## Results

### Antibiotic production potential of the fungi isolates

The result of the overlay experiment which was used to determine which fungi would be selected for identification and further work is available on request. F6 was selected based on the observed ‘broad’ spectrum of activity and capability of secreting substances that inhibited the growth of most of the test organisms.

### Identification of selected fungi isolate

The fungus F6 was identified to be *Fusarium solani* using a combination of molecular methods that comprised DNA isolation, PCR amplification, sequencing of the ITS region and nucleotide sequence blasting carried out in the laboratory of the Institute of Pharmaceutical Biology, Heinrich Heine University, Dusseldorf, Germany. The Query cover of the blast was 100% and the identification was also returned to be 100% certain. The DNA sequence has been deposited in GenBank and the GenBank accession number is MG772815.

### HPLC analysis of the extract

**Figure 1.**
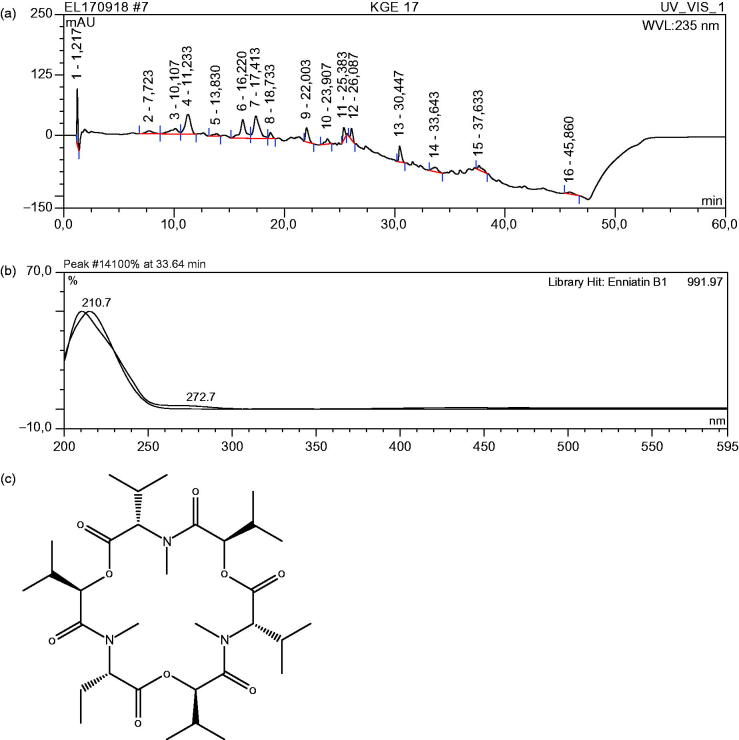
(a) Chromatogram of *Fusarium solani* extract at 235 nm. (b) UV spectra of peak 14. (c) Structure of enniantin, a cyclohexadepsipeptide.

HPLC analysis of the extract resulted in the detection of 17 distinct peaks representing at least 17 compounds (Figure 1(a)). One of the peaks (no. 14) was identified *via* dereplication to be enniantin because the database used contained data that matched its UV spectra. The remaining 16 peaks had no acceptable matches based on the algorithm of the dereplication software. The UV spectrum of enniantin are shown in Figure 1(b) and the structure in Figure 1(c). The HPLC analysis of the extract provided initial information of the number of compounds in the extracts that were UV active since the detection was carried out with Diode Array UV Detector. The enniantin that was identified was highly conjugated which is one of the characteristics of compounds that are UV active.

### Xylene induced oedema model

The anti-inflammatory potential of the extract was investigated using xylene model. The ability of the extract to inhibit xylene-induced inflammation which was comparable to that of diclofenac is shown in Table 1. A dose-dependent inhibition was observed for the extract and its activity at 500 µg/mL was stronger than that of diclofenac for topical application at 250 µg/mL, indicating a potential for use as an anti-inflammatory agent.

**Table 1.** The anti-inflammatory activity of *Fusarium solani* extracts *in vivo* using xylene model.

Treatment	Mean ear weight (mg)(left ear) ± SEM	Mean ear weight (mg)(right ear) ± SEM	%Inhibition
Extract 250 µg	15.2 ± 2.12	10.8 ± 0.20	19.0134
500 µg	15.7 ± 1.8	13.8 ± 0.18	65.635*
Diclofenac 500 µg	20.0 ± 0.5172	8.9 ± 0.39	61.807
10 µL of ethanol	18.2 ± 1.22	12.8 ± 1.35	0

*Not significantly different from the positive control.

### Fresh egg albumin induced rat paw oedema model

The anti-inflammatory property of the extract was further investigated in a rat paw inflammation model using fresh egg albumin as the phlogistic agent to further establish that the extract has significant anti-inflammatory potential (Table 2). Similar to the xylene model, the extract exhibited time-dependent activity that was significantly better than diclofenac in the first hour especially at 500 µg/mL. This confirmed the anti-inflammatory activity of extract and gave more insight as to the kinetics of the interaction.

**Table 2.** The %inhibition of egg albumin-induced rat paw oedema by *F. solani* extract.

Test samples	Dosage	%Inhibition of oedema formation with time				
		30 min	1 h	2 h	3 h	4 h
Control	10 mL/kg distilled water	0	0	0	0	0
Diclofenac	1.66 mg/kg diclofenac	9	8	36	57.45	85
F6 Extract	250 mg/kg	0	0	21.91	7.4	1.14
	500 mg/kg	16.59**	14.89**	39.36	21.91	24.54

**Significantly different from the positive control.

### Acetic acid-induced stomach writhing

Some compounds that possess anti-inflammatory properties have been known to have analgesic property which is the case with non-steroidal anti-inflammatory agents (NSAIDs). Therefore, the analgesic property of the extract was tested using acetic acid-induced writhing model to determine the dose of the extract that was able to inhibit acetic acid-induced writhing compared to untreated group. As shown in Table 3, the analgesic property of the extract was found to be inferior compared to diclofenac with a lower percentage inhibition that was not dose-dependent.

**Table 3.** Analgesic effect of *F. solani* extracts on the acetic acid-induced writhing in mice.

Test samples	Dosage	Mean number of writhings ± SEM	%Inhibition of writhings
Tween 80	10 mL/kg	33.67 ± 4.33	0
Diclofenac	15 mg/kg	9.33 ± 4.98	72.29
F6 Extract	250 mg/kg	17.0 ± 6.66	49.51
	500 mg/kg	24.67 ± 8.51	26.73

### COX-1 and 2 colourimetric inhibition assay

**Figure 2.**
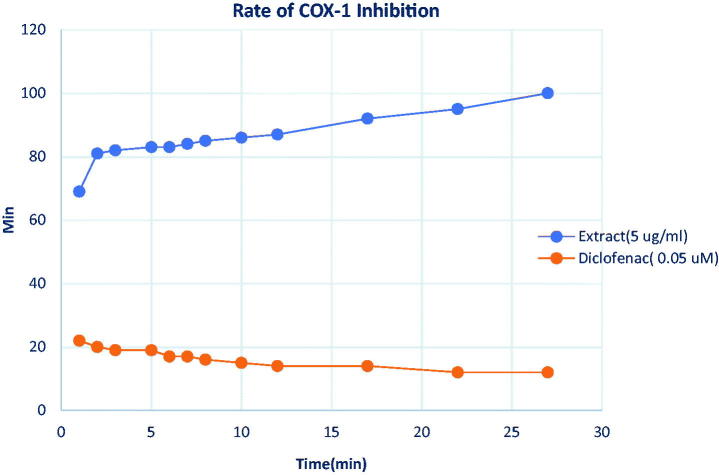
Time-dependent inhibition of COX-1 enzyme by *F. solani* extract.

The result of the COX-1 and COX-2 inhibitory assay showed that the extract had a potent inhibitory activity against COX-1 while it had no activity against COX-2 enzyme. The concentrations of the extract used for the test were within the range recommended by the manufacturers (30 and 100 µg/mL). Considering the addition of 180 µL (150 µL buffer, 10 µL Heme and 20 µL AA, 10 µL enzyme) to the reaction mixture, the final concentrations of the extract would be divided by 20 which will give 5 µg/mL for an initial dose of 100 µg/mL of the extract and 0.05 µM for diclofenac. The IC_50_ of the extract against COX-1 is below 5 µg/mL (Table 4). Table 4 shows the percentage inhibition values for COX-1 activities at different time points for the extract at different doses. The positive control and the extract at 5 µg/mL were also represented graphically (Figure 2).

**Table 4.** Time-dependent inhibition (%) of COX-1 enzyme activity by *F. solani* extract *in vitro*.

		Percentage inhibition/time											
Time (min)	1	2	3	5	6	7	8	10	12	17	22	27	32
F6 25 μg	−8	−9	−9	−9	−9	−10	−9	−9	−9	−10	−10	−10	−9
50 μg	−36	−34	−29	−27	−26	−26	−25	−24	−23	−23	−21	−19	−18
100 μg	69	81	82	83	83	84	85	86	87	92	95	100	104**
Diclofenac 1 µM	22	20	19	19	17	17	16	15	14	14	12	12	11

**Significantly different from the positive control for all figures in the row.

### Determination of MIC of the extract against test organisms

The MIC of the extract against selected test pathogens was determined. The result shows that the extract showed MIC <12.5 µg/mL for *S. aureus*, *E. coli* and *P. aeruginosa* whereas it showed higher MICs for other organisms tested. This indicates that the extract also has reasonable antibacterial property.

## Discussion

The results of the different tests carried out on the ethyl acetate fermentation extract of *F. solani* showed that it possesses potent antibacterial and anti-inflammatory properties. In addition, the extract strongly inhibited COX-1 enzyme in *in vitro* experiment.

The antimicrobial property of the extract was profound against *P. aeruginosa*, *E. coli* and *S. aureus*. The MICs of the extract against these organisms were below 12.5 µg/mL while the MICs for *B. subtilis* and *S. typhi* were 25 and 100 µg/mL, respectively, indicating a broad range of activity against both Gram-positive and Gram-negative organisms. Although the MIC of the extract against *S. typhi*, one of the three Gram-negative organisms, was 100 µg/mL, considering that Gram-negative organisms have innate resistance capability because of the characteristic nature of their cell membrane, this result is of significant interest and importance. The extract appeared to have predominantly oily texture; therefore, it could be that its oily nature facilitates its passage or interaction with the hydrophobic cell membrane of the Gram-negative organisms. It is worthwhile to mention that no new growth was observed on the plates even after 7 d of further incubation suggesting that the observed absence of growth was due to bactericidal activity of the extract rather than bacteriostatic activity. Our result agrees with the report by Kyekyeku et al. (2017) on the antibacterial activity of *F*. *solani* extract and compounds suggesting that the Gram-negative bacteria are very susceptible to *Fusarium solani* metabolites.

The anti-inflammatory property of the extract was also evaluated using two established inflammation models. The xylene-induced ear oedema model was used to evaluate the ability of the extract to inhibit topically induced acute inflammation while the rat paw oedema model was used to study its activity against systemically induced inflammation. Topical application of xylene instantly results in the increase in vascular permeability as a response to the irritation caused by the chemical. The increased vascular permeability allows the infiltration of fluid and protein into the extravascular area resulting in oedema which is a characteristic of acute inflammation (Atta and Alkohafi 1998; Ono et al. 2017). Although the extract (500 µg/mL) was able to inhibit this process by 65% compared to the 61% observed for the positive control, diclofenac (500 µg/mL), there was no significant difference *p* < 0.05 between their effects. There was, however, a significant difference between the effects of the extract at 500 µg/mL compared to its effect at 250 µg/mL. This suggests that the extract has the ability to inhibit acute inflammation in a dose-dependent manner. Oedema model induced by xylene has been widely used for estimating the anti-inflammatory properties of potential drug candidates because of its excellent predictive values (Ma et al. 2013; Sowemimo et al. 2013).

The dose range of the extract determined from the xylene model was applied for the egg albumin model. Inflammation induced by egg albumin is reported to be biphasic. The first phase that occurs within 2 h of inflammation involves the release of histamine and serotonin that results in an increased vascular permeability whereas the second phase is mediated by the prostaglandins, the kinins and the lysosomes (Anosike et al. 2012; Ma et al. 2013). The effect of the extract at 250 mg/kg was not significantly (*p* < 0.05) better than that of the negative control against the development of inflammation for the first 1 h. However, the anti-inflammatory activity of the extract at this dose reflected afterwards as could be seen in the percentage inhibition of oedema formation which peaked after 2 h. After that, the percentage of inhibition decreased with time. At 500 mg/kg, the extract demonstrated a rapid onset of inhibition of oedema formation significantly (*p* < 0.05) better than the effect of the positive control, diclofenac (Table 4). Similar to the effect observed at 250 mg/kg, the effect of the extract at 500 mg/kg peaked at 2 h and afterwards reduced with time. The extract at 500 mg/kg was significantly (*p* < 0.05) better than at 250 mg/kg as well as the negative control which confirms its dose-dependent activity as was observed for the xylene model. The early onset of action suggests that this extract could be very useful for acute inflammation and if in combination with an agent whose activity peaks in the second phase, for example diclofenac, could potentiate its effect in the management of inflammatory processes. It has been reported that NSAIDs do not effectively inhibit the first phase of inflammation (Salvemini et al. 1996). This explains the reason why the percentage inhibition observed for diclofenac within the first 1 h was in single digits. The reduced activity in the second phase for the extract also suggests that the extract may have little or no direct effect on COX site reaction, especially for COX-2 isoform, whose upregulation leads to the recruitment and production of inflammatory mediators, the presence of which characterize the second (cellular) phase of inflammation (Ricciotti and Fitzgerald 2011). Rat paw oedema model is reliable and of good value in studying the contribution of inflammatory mediators in inflammatory response (Salvemini et al. 1996), therefore, it is widely used in the estimation of the anti-inflammatory potential of new drug candidates (Karakus et al. 2013; Cong et al. 2015).

Inflammatory mediators and consequently inflammation have been linked with pain (Dubin and Patapoutian 2010; Ronchetti et al. 2017). Often, compounds with anti-inflammatory activities alleviate pain. The extract was tested for its ability to modulate pain perception using the acetic acid-induced abdominal constriction model. The extract showed certain level of analgesic properties which, however, was not dose dependent within the dose range tested (Table 3). The observed analgesic property which was estimated based on the ability of the agent to inhibit abdominal constriction induced by acetic acid at 250 mg/kg was about 2/3 of the effect achieved by the positive control, diclofenac at 15 mg/kg. This result which is significantly better than the negative control (*p* < 0.05) deserves further consideration.

The mechanism of anti-inflammatory action was investigated *in vitro* to determine if the extract has any activity against COX enzyme isoforms 1 and 2. The rate of reaction between the enzyme and the extract was monitored for over 30 min. It was found that the extract at the dose of 5 µg/mL inhibited 69% of the enzyme activity after 2 min. The inhibition increased with time and reached 100% before 30 min. On the other hand, the positive control at the final dose of 0.05 µM exhibited an initial enzyme inhibition of 22% which was slightly decreased towards the end of the 30 min period. The effect of the extract was significantly better than that of the positive control at *p* < 0.05. NSAIDs are known to bind slowly, competitively and reversibly to COX enzyme (Meek et al. 2010) and as a result, their inhibition of the enzyme is not maintained for a long period of time. This could explain the reduction in the inhibition percentage of the enzyme with time. Also, classical NSAIDs preferentially target the COX reactions rather than the peroxidase reaction (Szewczuk et al. 2004; Bettelheim et al. 2012) . On the other hand, neither the extract nor the positive control had an inhibitory effect on COX-2 in this investigation. Because classical NSAIDs do not significantly inhibit peroxidase reactions (Bettelheim et al. 2012), the inhibition percentage observed for diclofenac (the positive control) in this peroxidase-based assay was justified. Some compounds, such as resveratrol known to inhibit both isoforms of COX have been reported to have weak effect on the peroxidase activity of COX-2 (Szewczuk et al. 2004). The inhibition of COX-1 catalytic activity is desirable in the prevention of cardiovascular conditions. The lack of inhibition of COX-2 enzyme activity by the extract further explains the reason for the reduced activity in the second phase of inflammation in rat paw oedema experiment.

The HPLC analysis of the extract and dereplication revealed one known compound, enniantin, a cyclic depsipeptide known to have antibiotic property (Zaher et al. 2015) which could have contributed to the profound antibiotic property observed. Although the antibacterial properties of endophytic strain of *F. solani* extracts and some compounds have been reported (Kyekyeku et al. 2017), we were able to demonstrate and provide the experimental evidence that the antibacterial property could be bactericidal. It could also be possible that the range of secondary metabolites from the two strains may differ even though both originated from West African countries of Ghana and Nigeria, respectively. Also, while our source was the soil around a power generating plant house, which is considered to be a stress area, the other was an endophyte from a plant part collected from the forest. The differences pointed out could have generated different types of metabolic pathways suitable for survival in their respective environments. It is possible that the antibacterial properties are not mediated by the same compounds.

## Conclusions

In addition to the antimicrobial properties of the *Fusarium solani* extract, we are also reporting for the first time that the anti-inflammatory property is concomitantly present. Different *in vivo* models of inflammation were used to study the anti-inflammatory potentials of the extract and all suggested that the extract possesses strong anti-inflammatory property especially at the first phase of inflammation. Our findings also suggested that the extract does not inhibit COX-2 activity but in combination with other NSAIDs could result in a potent arsenal against inflammatory conditions. The extract also exhibited profound antibacterial activity against Gram-negative organisms used in the test. A combination of antimicrobial and anti-inflammatory property in a single extract can find many applications in the pharmaceutical industry.
